# Multicenter prospective validation study of the SAFE-T colonoscopy evaluation tool: a web-based smartphone application for evaluation of gastroenterology fellow performance in colonoscopy

**DOI:** 10.1093/gastro/goaa063

**Published:** 2020-10-04

**Authors:** Navin L Kumar, Guillaume Kugener, Abhinav Vemula, Sharmeel K Wasan, Amiko Uchida, Fabian J Scheid, Joseph Yarze, John R Saltzman

**Affiliations:** 1 Division of Gastroenterology, Hepatology and Endoscopy, Brigham and Women’s Hospital, Boston, MA, USA; 2 Harvard Medical School, Boston, MA, USA; 3 Massachusetts Institute of Technology, Cambridge, MA, USA; 4 Division of Gastroenterology, Boston Medical Center, Boston, MA, USA; 5 Boston University School of Medicine, Boston, MA, USA; 6 Division of Gastroenterology, Massachusetts General Hospital, Boston, MA, USA

## Introduction

One of the key objectives of gastroenterology (GI) fellowships is to teach fellows how to perform high-quality endoscopic procedures [1]. The primary model for this education is the apprenticeship approach, in which fellows perform endoscopic procedures on patients under the supervision of an attending gastroenterologist [2]. Critical to the success of this teaching model, faculty should deliver timely and actionable feedback based on direct observation of the fellow’s endoscopic performance [3]. This need for feedback is particularly true for fellows learning how to perform colonoscopy, which requires additional experience to gain competency compared with upper endoscopy [4].

To aid in the delivery of this feedback, a variety of assessment tools have been validated in the trainee population, including the Mayo Colonoscopy Skills Assessment Tool (MCSAT) that was later refined into the Assessment of Competency in Endoscopy (ACE) tool for colonoscopy [5, 6]. However, both tools are paper-based and have multiple questions to complete, which limit their use as a continuous assessment tool. At our institution, we previously developed and validated the Skill Assessment in Fellow Endoscopy Training (SAFE-T) tool as a concise five-question evaluation tool that is administered via a web-based application and showed excellent correlation to the MCSAT overall hands-on and individual motor scores [7]. One of the major limitations of that study, however, was that it was conducted at a single GI fellowship program with unclear generalizability to other sites. Our aim in this investigation was therefore to validate the SAFE-T colonoscopy tool in a multicenter study.

## Methods

### Assessment tool

The SAFE-T tool captures both summative and formative feedback on a fellow’s individual performance during colonoscopy (Supplementary Table 1) [7]. In addition to assessing objective performance and case complexity, the tool includes an overall-performance score that ranges from 1 (beginner) to 5 (superior) with qualifiers based on need for hands-on assistance and/or coaching. The final question requests faculty to identify a single area of improvement for the fellow based on their colonoscopy performance.

### Study participants

All general GI fellows (Years 1–3 of training) at three academic ACGME-accredited GI fellowship programs in Boston, Massachusetts were eligible to participate. Attending gastroenterologists who supervise fellows performing colonoscopy were eligible to participate in the study as evaluators. The study was conducted over a 10-month period from July 2018 (start of the academic year) through the end of April 2019. The study was reviewed by the Institutional Review Board at Partners Healthcare and was given exempt status.

### Data collection and analysis

We adapted the SAFE-T colonoscopy tool into a web-based application that was optimized for use on smartphones and computers. Each faculty member had a unique login and password to access the application. Completed SAFE-T evaluations were automatically logged and transmitted to a password-protected central repository. To assess how the SAFE-T colonoscopy tool correlated with other relevant variables, we used independent *t*-tests to compare the mean SAFE-T overall-performance score across (i) trainee year, (ii) case complexity (by insertion) and (iii) successful vs failed cecal intubation. *P*-values <0.05 were considered significant. All analyses were conducted using R version 3.5.2 (R Core Team, 2018).

## Results

**Figure 1.**
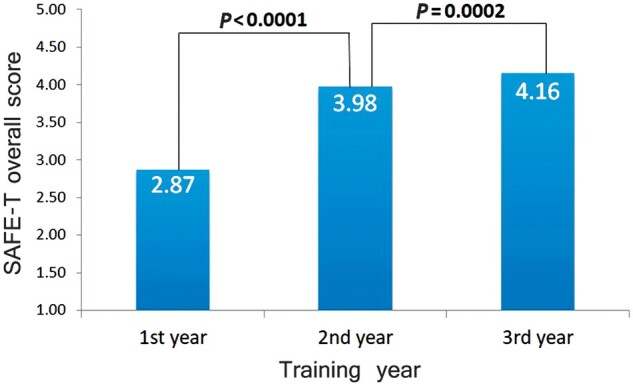
Skill Assessment in Fellow Endoscopy Training (SAFE-T) overall scores by training year.

During the study period, 39 endoscopy faculty used the SAFE-T colonoscopy tool to complete 1,249 evaluations of the 37 fellows. First-year fellows (*n *=* *12) completed 183 procedures (14.6%), second-year fellows (*n *=* *12) completed 533 procedures (42.7%), and third-year fellows (*n *=* *13) completed 533 procedures (42.7%). The mean SAFE-T overall score increased with each sequential fellow year of training (Figure 1), with second-year fellows having significantly higher scores compared with first-year fellows (3.98 vs 2.87, *P *<* *0.0001) and third-year fellows having significantly higher scores compared with second-year fellows (4.16 vs 3.98, *P *=* *0.0002). The mean SAFE-T overall score decreased with increasing case-complexity score, with average cases having significantly lower scores compared with straightforward cases (3.81 vs 4.24, *P *<* *0.0001) and challenging cases having significantly lower scores compared with average cases (3.35 vs 3.81, *P *<* *0.0001). Cases with successful cecal intubation had significantly higher SAFE-T overall scores than cases that did not reach the cecum (4.24 vs 2.57, *P *<* *0.0001).

## Discussion

In this multicenter prospective study of GI fellows, our results demonstrate the validity across multiple ACGME-accredited institutions of the SAFE-T colonoscopy-evaluation tool. Adapted for smartphone use, this tool allowed point-of-care assessment and encouraged faculty to provide feedback after each supervised colonoscopy via a web-based application. The results of this study helped to confirm the findings of our prior single-center SAFE-T investigation [7]. As in our original study, the SAFE-T overall-performance score improved with increased fellow experience and in cases with successful cecal intubation while declining in cases of increasing complexity. These multicenter results further validate the utility of the SAFE-T colonoscopy tool and demonstrate its generalizability.

As an assessment tool that is concise with just five questions to complete, the SAFE-T tool does not include detailed performance data such as that generated by the more comprehensive ACE colonoscopy tool. However, the shorter nature of the form and its adaptation into a web-based application allows endoscopy faculty to readily use the tool in routine clinical practice after each supervised colonoscopy. The SAFE-T colonoscopy tool may thus be used for continuous assessment of a trainee’s endoscopic skills with the ACE colonoscopy tool completed at regular intervals (e.g. every 50th procedure) in a combined approach to provide a comprehensive assessment of fellow endoscopic skills.

The study does have some important limitations. First, the faculty was not blinded to the fellow in each supervised case and may have been biased by knowledge of the fellow’s year of training or past endoscopy performance. However, given the apprenticeship nature of endoscopic training, blinding would not be feasible, nor would it be applicable to real-world endoscopic training. Second, one of our study sites did not use electronic endoscopy software to allow tracking of cases and thus we were unable to calculate a response rate for the SAFE-T colonoscopy tool across the multiple sites. Third, the concise nature of the tool did not allow the rating of specific endoscopic skills. However, the last question of the SAFE-T tool does identify specific skills for improvement by the fellow.

In conclusion, we demonstrated the validity and generalizability of the SAFE-T colonoscopy tool in a multicenter prospective study. The continuous data generated from the tool allow GI fellows to individually track their performance over time, endoscopy faculty to continually provide feedback on colonoscopy skills and tailor teaching to specific fellows based on prior evaluations, and fellowship programs to track the endoscopic performance of GI fellows and provide focused feedback to individual fellows throughout their training.

## Supplementary data

Supplementary data is available at *Gastroenterology Report* online.

## Funding

This work was supported by the Clinical Education Research Scholars Program of the Department of Medicine at Brigham and Women’s Hospital (award recipient—Navin L. Kumar)

## Supplementary Material

goaa063_Supplementary_Data
