# Supplementary material for: Multicenter prospective validation study of the SAFE-T colonoscopy evaluation tool: a web-based smartphone application for evaluation of gastroenterology fellow performance in colonoscopy
Source: Gastroenterol Rep (Oxf). 2020 Oct 4;9(2):176–8. doi: 10.1093/gastro/goaa063 (PMC8128019; doi:10.1093/gastro/goaa063)
Supplement: goaa063_Supplementary_Data [file goaa063_supplementary_data.pdf]

Supplementary Table 1. Skill Assessment in Fellow Endoscopy Training (SAFE-T) colonoscopy form.

Farthest landmark reached (without any hands-on assistance)
1. Rectum
2. Sigmoid colon
3. Descending colon
4. Transverse colon
5. Ascending colon
6. Cecum
7. Terminal ileum
Complexity of Insertion
1. Straightforward (no pressure or position change needed)
2. Average (some pressure or one position change needed)
3. Challenging (significant pressure and multiple position changes needed)
Complexity of Intervention(s)
0. N/A (no intervention performed)
1. Straightforward (biopsy)
2. Average (snare polypectomy)
3. Challenging (complex polypectomy)
Overall Performance
1. Beginner (significant hands-on assistance and coaching)
2. Advanced beginner (some hands-on assistance and/or significant coaching)
3. Intermediate (limited hands-on assistance and/or some coaching)
4. Proficient (no hands-on assistance but needs extra time)
5. Superior (able to perform exam independently and efficiently)
For the next case, the fellow should focus on improving this one aspect:
• Pre-procedure evaluation (indication, comorbidities, etc.)
• Patient discomfort monitoring and management
• Lumen identification
• Loop management
• Safe endoscopic advancement
• Cecal intubation
• Terminal ileum intubation
• Visualized mucosa during withdrawal
• Pathology identification
• Biopsy technique
• Snare polypectomy technique
• Rectal retroflexion
• N/A (no specific area to improve)
